# Identification of a Human *SOCS1* Polymorphism That Predicts Rheumatoid Arthritis Severity

**DOI:** 10.3389/fimmu.2020.01336

**Published:** 2020-06-26

**Authors:** Amalia Lamana, Ricardo Villares, Iria V. Seoane, Nuria Andrés, Pilar Lucas, Paul Emery, Edward M. Vital, Ana Triguero-Martínez, Ana Marquez, Ana M. Ortiz, Robin Maxime, Carmen Martínez, Javier Martín, Rosa P. Gomariz, Frederique Ponchel, Isidoro González-Álvaro, Mario Mellado

**Affiliations:** ^1^Rheumatology Service, Instituto de Investigación Sanitaria La Princesa, Hospital Universitario de la Princesa, Madrid, Spain; ^2^Department of Immunology and Oncology, Centro Nacional de Biotecnología/CSIC, Madrid, Spain; ^3^Department of Cellular Biology, Facultad de Biología, Universidad Complutense de Madrid, Madrid, Spain; ^4^Leeds Institute of Rheumatic and Musculoskeletal Medicine (LIRMM), The University of Leeds, Leeds, United Kingdom; ^5^Institute of Parasitology and Biomedicine López-Neyra, CSIC, Granada, Spain

**Keywords:** rheumatoid arthritis, disease activity, cytokines, inflammation, biomarkers

## Abstract

Rheumatoid arthritis (RA) is a chronic inflammatory disease characterized by an autoimmune response in the joints and an exacerbation of cytokine responses. A minority of patients with RA experience spontaneous remission, but most will show moderate/high disease activity, with aggressive joint damage and multiple systemic manifestations. There is thus is a great need to identify prognostic biomarkers for disease risk to improve diagnosis and prognosis, and to inform on the most appropriate therapy. Here we focused on suppressor of cytokine signaling 1 (SOCS1), a physiological negative regulator of cytokines that modulates cell activation. Using four independent cohorts of patients with arthritis, we characterized the correlation between *SOCS1* mRNA levels and clinical outcome. We found a significant inverse correlation between *SOCS1* mRNA expression and disease activity throughout the follow-up of patients with RA. Lower baseline *SOCS1* levels were associated with poorer disease control in response to methotrexate and other conventional synthetic disease-modifying anti-rheumatic drugs in early arthritis, and to rituximab in established (active) RA. Moreover, we identified several single nucleotide polymorphisms in the *SOCS1* gene that correlated with *SOCS1* mRNA expression, and that might identify those patients with early arthritis that fulfill RA classification criteria. One of them, rs4780355, is in linkage disequilibrium with a microsatellite (TTTTC)_3−5_, mapped 0.9 kb downstream of the SNP, and correlated with reduced *SOCS1* expression *in vitro*. Overall, our data support the association between *SOCS1* expression and disease progression, disease severity and response to treatment in RA. These observations underlie the relevance of *SOCS1* mRNA levels for stratifying patients prognostically and guiding therapeutic decisions.

## Introduction

Rheumatoid arthritis (RA) is a systemic autoimmune disease characterized by chronic inflammation in diarthrodial joints. RA is known to have a very heterogeneous clinical course with a period of preclinical disease, and can be classified in several phases, from the initial genetic and environmental risk factors for RA, to the development of autoimmunity, arthralgia symptoms and inflammatory arthritis, and finally to established RA (1).

A small number of patients with inflammatory arthritis experience spontaneous resolution of symptoms, and some have a mild disease course with slow progression, which is diagnosed as undifferentiated arthritis (UA). Most patients with inflammatory arthritis nonetheless fulfill the *European League Against Rheumatism* (EULAR) 2010 criteria for RA and develop moderate-to-high disease activity and aggressive joint damage, often with systemic complications. In this scenario, clinical trials (2, 3) and daily clinical practice (4) have confirmed that early treatment with disease-modifying anti-rheumatic drugs (DMARDs) improves the outcome patients with early RA. To avoid the generalized use of aggressive therapies that could expose patients to unjustified risks of side effects and whose cost would needlessly burden National Health Services, much effort has been made to identify prognostic biomarkers for disease risk, to improve diagnosis and prognosis and to help guide the most appropriate therapy for each patient (5).

Cytokines were originally identified as soluble messengers in the context of the immune system, but are now known to be released by myriad cell types with multifunctional actions on cell proliferation, survival, apoptosis, differentiation and activation. Based on their role in the immune response, cytokines are classified as either pro- or anti-inflammatory (6), and some have key functions in different stages of RA (7). Cytokines act by engaging specific receptors constitutively associated with the Janus family tyrosine kinase–signal transducer and activator of transcription (JAK–STAT) signaling pathway. Upon ligand binding to receptors, JAKs are activated by transphosphorylation, and phosphorylate both the receptor and members of the STAT family of transcription factors. In turn, activated STATs dimerize and translocate to the nucleus to regulate gene expression (8). Dysregulated activation of the JAK–STAT pathway is associated with many diseases, including autoimmune disorders such as RA (9).

Cytokine-mediated signaling pathways are controlled precisely at several levels, including activation of phosphatases, upregulation of proteins that interfere with STAT binding to DNA, and expression of SOCS (suppressor of cytokine signaling) proteins, which suppress JAK activity, prevent STAT recruitment to the receptor, and induce substrate degradation (10–12). The SOCS proteins constitute a family of intracellular proteins comprising eight members that, among other processes, regulate cytokine-triggered signaling and cell activation status (13). While a recent genome-wide meta-analysis failed to identify a significant association between RA and *SOCS1* (14), *SOCS1* mRNA levels are significantly increased in peripheral blood T-cells and in synovial membranes of RA patients as compared with patients with osteoarthritis (15), pointing to a possible role for SOCS1 in RA.

In the present study, we used four independent cohorts of patients with arthritis to test for associations between *SOCS1* expression and RA. We observed an inverse correlation between *SOCS1* mRNA expression levels and disease activity, with lower baseline *SOCS1* levels associating with poorer disease control. Genotyping analysis identified several single nucleotide polymorphisms (SNPs) in the *SOCS1* gene that associate with RA development and with response to treatment. Finally, *in vitro* expression analysis indicated that the minor allele of one of these SNPs, rs4780355, correlated with reduced *SOCS1* expression, supporting a functional relationship between this SNP and disease progression.

## Patients, Materials, and Methods

### Study Populations

#### Princesa Early Arthritis Register Longitudinal (PEARL) Study

The PEARL study includes patients referred to the Early Arthritis Clinic at the Hospital Universitario La Princesa, Madrid (Spain) (16). Inclusion criteria were at least one swollen joint and symptoms for <1 year. The register protocol included the collection of socio-demographic, clinical, and therapeutic data, as well as samples obtained in four scheduled visits (baseline, 6, 12, and 24 months). At the 24-month visit, a diagnosis of RA was established definitively based on the 1987 *American College of Rheumatology* criteria (17) as opposed to chronic UA (18) (Table 1). The PEARL study was approved by the Ethics Committee for Medical Research (CEIM, Hospital Universitario La Princesa. Instituto de Investigación Sanitaria La Princesa, Madrid; PI-518). All patients gave written informed consent. From the PEARL register, we selected both discovery and validation populations (Supplementary Table 1, Studied), The discovery group included patients that did (RA) or did not (UA) fulfill RA criteria at the end of follow-up, whereas the validation group included more stringent criteria for inclusion and all patients fulfilled the 1987 RA criteria at the end of follow-up. The main characteristics of the two groups are shown in Supplementary Table 2.

**Table 1:** Baseline characteristics of the populations studied.

	**PEARL study (*n =* 456)**	**LEEDS (*n =* 74) early RA**	**LEEDS (*n =* 64) established RA**
Female; n (%)	361 (79%)	56 (76%)	52 (81%)
Age^*^ (years)	55 (44–67)	53 (43–73)	58 (48–67)
Disease duration (months)^*^	5.4 [3.0–8.5]	6.5 (4–13)	84 (36–204)
RF positive; n (%)	243 (53%)	37 (50%)	64 (100%)
ACPA positive; n (%)	220 (49%)	44 (60%)	64 (100%)
DAS28^*^	4.5 [3.4–5.6]	4.3 (3.15–5.4)	5.7 [4.8–6.3]
HAQ^*^	1 [0.5–1.625]	na	na

n, number;

* *median and interquartile range; RA, rheumatoid arthritis; RF, rheumatoid factor; ACPA, anti-citrullinated protein antibodies; DAS28, disease activity score calculated with the 28 joint count; HAQ, health assessment questionnaire; na, not available*.

#### Leeds Cohorts

Leeds patients (Table 1) were included in two cohorts. The first cohort included 74 DMARD-naïve patients with early RA fulfilling the 2010 EULAR criteria that were selected from a prospective Early Arthritis Register (Table 1) and were treated in a standardized fashion with methotrexate at 15 mg/week, increasing to 25 mg/week over 8 weeks if remission was not achieved. Additional DMARDs (sulfasalazine or hydroxychloroquine) were used if low disease activity was not achieved by 3 months. The DAS28 (Disease Activity Score including a 28-joint count) was used as a 6-month outcome to classify response, with a DAS28 score <2.6 defining remission. The second cohort included 64 patients with established RA who showed an inadequate response to classic/synthetic DMARD treatment and were treated with rituximab (Table 1). Approval for the study with the Leeds cohorts was obtained from the North East Yorkshire Research Ethics Committee (PI: 07/S0703/68, 10/H1307/138). Written informed consent was obtained from all patients.

### DNA Extraction, qRT-PCR, and PCR

For all samples, peripheral blood mononuclear cells (PBMCs) were isolated by Ficoll density gradient centrifugation (Histopaque-1077, Sigma-Aldrich, USA). For the PEARL study samples, total RNA (2 μg) obtained using the TRI Reagent (Sigma-Aldrich) was reverse-transcribed using the High-Capacity cDNA Reverse Transcription Kit (Applied Biosystems, USA). For the Leeds samples, RNA was extracted using a standard guanidium/phenol method and first-strand cDNA was synthesized using a High-Capacity cDNA Reverse Transcription Kit (Thermo Fisher, USA).

For *SOCS1* analysis, and in order to confirm an amplification efficiency ~2, three 1:4 serial dilutions of each cDNA (corresponding to ~5 ng, 1.25 ng and 0.31 ng of the original RNA per well) were amplified in triplicate by qRT-PCR on an ABI Prism HT7900 sequence detection system using SYBR Green PCR Master Mix (both from Applied Biosystems) and 0.3 μM of the following primers:

*SOCS1*.5′: ACCCCGTCCTCCGCGACTAC; *SOCS1*.3′: TCCGCTCCCTCCAACC CAGG; β*-actin*.5′: AGCGAGCATCCCCCAAAGTT; β*-actin*.3′: GGGCACGAAGGCT CATCATT; *GAPDH*.5′: AGAAGGCTGGGGCTCATTTG; *GAPDH*.3′: AGGGGCCA TCCACAGTCTTC; *HPRT1*.5′: ACCAGTCAACAGGGGACATAAAAG; *HPRT1*.3′: GTCTGCATTGTTTTGCCAGTGTC. For relative quantification, the higher template concentration (5 ng) of each sample was used, normalized against the expression of *ACTB* and with the mean value obtained from 40 healthy donors (2^−ΔΔCt^) (19). *GAPDH* was used as a second internal control to validate *ACTB* levels. The same primers were used for *SOCS1* in samples from the Leeds study, and *HPRT* was used as a housekeeping gene: *HPRT*.5′: GGAAAGAATGTCTTGATTGTGGAAG; *HPRT*.3′: AAGGAACCAGTCC GTCATATTAGG.

For *in vitro* phytohemagglutinin (PHA; Sigma, Spain) activation assays, *ACTB* and *HPRT1* were used in a similar manner, although in this case normalization against *HPRT1* levels was used for the figures shown here.

*SOCS1* expression in the PEARL-validation group was analyzed using the Roche RealTime ready Custom Panels 384 and LightCycler 480 Probes Master with pre-plated primers from a Universal Probe Library (Roche, Germany). Assays were performed in triplicate and results were normalized to *ACTB* expression levels.

### SOCS1 Genotyping

The Immunochip array on the Illumina iScan System (Illumina Inc., USA) was used to genotype 261 patients from the PEARL study. The chip array includes 196,000 SNPs with genetic positions according to the NCBI build 36 (hg18) map (Illumina manifest file Immuno_BeadChip_11419691_B.bpm). To control batch effects, stringent quality controls were applied to datasets prior to the final analysis. Raw data from Immunochips were filtered using PLINK v1.07 software as follows: low-quality SNPs were discarded when call rates were <95%, minor allele frequencies (MAFs) <0.01, and deviation from the Hardy-Weinberg equilibrium *p* < 0.001. Samples were also discarded when they showed <90% of successfully called SNPs. Ten SNPs were selected for subsequent genotyping in the 571 remaining PEARL early arthritis patients. The selected *SOCS1* genetic variants (rs11074956, rs181582, rs149597, rs2021760, rs4780355, rs193779, rs243327, rs1559392, rs3844576, and rs243323) were genotyped using predesigned TaqMan probes (Applied Biosystems, assay ID: C___3189858_10, C___1004298_10, C___3189853_10, C__11185228_10, C___3189846_10, C___1004284_10, C___3189840_10, C___9697634_10, C___3189819_10, C___3189829_10, respectively). After PCR, the genotype of each sample was attributed automatically by measuring allele-specific fluorescence on a CFX Touch Real-Time PCR System using CFX 3.1 Manager (BioRad, USA). Duplicate samples and negative controls were included to verify genotyping accuracy.

### PBMC Activation

To resolve the problem of the small volume of available blood obtained from patients, we first determined the optimal experimental conditions to activate PBMCs from both healthy donors and patients with RA. PBMCs were isolated as described and were activated with PHA (5 μg/ml). A peak of *SOCS1* transcription was detected between 2 and 4 h post-stimulation, followed by a decline and then a second peak at ~16 h. We included NF-kB as an activation marker, as it is well-known to regulate inflammatory genes and cytokine production (20). Because the second upregulation peak might be the result of responses to multiple cytokines secreted by the distinct PBMC populations, we selected the early stimulation peak for comparative analysis.

Thus, PBMCs from patients, healthy donors or, when required, buffy coats, were isolated as above, seeded at 10^6^ cells/ml, and stimulated with 5 μg/ml PHA for 3 h (21). Cells were then harvested for RNA extraction.

### Luciferase Assay

Genomic DNA that included the *SOCS1* polyadenylation signal and downstream sequences was cloned from DNA of buffy coats from healthy donors. Two clones with the rs4780355 minor allele (C) and two with the major allele (T) were selected for expression assays. A 790-base pair (bp) fragment comprising 68 bp upstream of the polyadenylation signal and rs4780355 (located 435 bp downstream), and a second fragment of 2.1 kbp with the same 5′ end and containing (TTTTC)_3−5_ located at 0.9 kbp 3′ of rs4780355, were PCR-amplified, individually cloned and sequenced verified. The fragments were cloned into the psiCHECK-2 vector (Promega, USA), between the *Renilla* luciferase coding sequence and its synthetic polyA site. The psiCHECK-2 plasmid has two different luciferase genes (Firefly and *Renilla*) driven by distinct promoters: the *Renilla* gene serves as a reporter, whereas the Firefly gene acts as an internal control. Plasmids were electroporated into Jurkat cells, which were cultured for 24 h (37°C, 5% CO_2_) and harvested. Cell lysates from 1–3 × 10^4^ cells were analyzed for *Renilla* and Firefly luciferase activity using the Dual Luciferase Reporter Assay System (Promega) and data were normalized to Firefly luciferase activity.

### Statistical Analysis

Statistical analyses were performed using Stata 14 for Windows (Stata Corp LP, USA) and Graphpad Prism 6 for Mac (GraphPad Software Inc., USA). Normally-distributed quantitative variables were expressed as the mean ± standard deviation, and non-normally-distributed variables were expressed as the median and interquartile range (IQR). In case of normal distribution, bivariate analyses were performed using a *t*-test; Mann-Whitney U, Kruskal-Wallis or *p*-trend tests were used for non-normally distributed variables. The χ^2^ test was used for qualitative variables.

#### Variables

*SOCS1* gene expression, expressed as 2^−ΔΔCt^, does not show a Gaussian distribution, and so data were normalized through logarithmic transformation. When required, a variable *SOCS1* baseline was used corresponding to low levels (low *SOCS1*), defined as those of samples from the baseline visit (PEARL or Leeds populations) below percentile 25 of their respective whole population.

Disease activity at the end of follow-up was defined by DAS28 cut-off values (22). In the case of the PEARL study, the EULAR response (22) was determined at visits at 6, 12, and 24 months relative to the baseline visit. A dichotomic variable “response” was developed for logistic regression by joining good and moderate EULAR responses.

Diagnosis at the end of follow-up in the PEARL population was expressed as a dichotomic variable between UA and RA as described above (see the Study Populations section).

#### Analyses

Cuzick's test was used to study *SOCS1* evolution during follow-up and its association with disease activity level.

As several variables can contribute to slight modifications in *SOCS1* expression, the descriptive analyses were followed by multivariate analyses based on generalized estimating equations (GEE) nested by patient and visit, using the *xtgee* command of Stata. This model allows a better-adjusted *SOCS1* value for each patient. Briefly, we used multivariate models by adding all variables with *p* < 0.20 in the bivariate analysis. This was followed by manual backward-stepwise selection to fit the final models by sequentially removing variables with *p* > 0.20, except the variable diagnosis that was forced since UA was included only in the discovery subpopulation.

To select the most relevant SNPs from the 10 identified as representative of genetic heterogeneity in the *SOCS1* region, we used three independent models of logistic regression with the following dependent variables: (a) low *SOCS1* expression at baseline visit adjusted by the relevant variables that affect *SOCS1* expression, selected according to the longitudinal multivariate model (age, diagnosis, glucocorticoid treatment, disease activity level, and hemoglobin); (b) diagnosis at the end of follow-up; and (c) therapeutic response at the 12-month visit, adjusted by the variables that affect DAS28 (age and sex) (23). As the studied population showed a very heterogeneous background, we performed a sensitivity analysis based on ethnicity (not shown). No differences were observed after this adjustment, indicating the ancestry of the population does not contribute to the expression of *SOCS1*, independently of the contribution of the SNPs described here.

## Results

### Patient Characteristics

Baseline characteristics of patients are listed in Table 1. In the PEARL register, 71% of patients fulfilled the criteria for RA at the end of 2-year follow-up, whereas 29% were considered to have UA. The Leeds population included 74 DMARD-naïve patients with early RA and 64 patients with established RA.

All PEARL study patients were genotyped for various SNPs in *SOCS1* (see Methods), but only a subpopulation (*n* = 143) with full follow-up and high-quality mRNA samples was used for *SOCS1* expression analysis (Supplementary Table 1; studied population). No significant differences were observed in the disease characteristics between patients studied and not studied, which indicates that the studied population is fully representative of the entire register (Supplementary Table 1).

### Low Baseline *SOCS1* Expression as a Biomarker of Disease Severity in Patients With Inflammatory Arthritis

**Figure 1:**
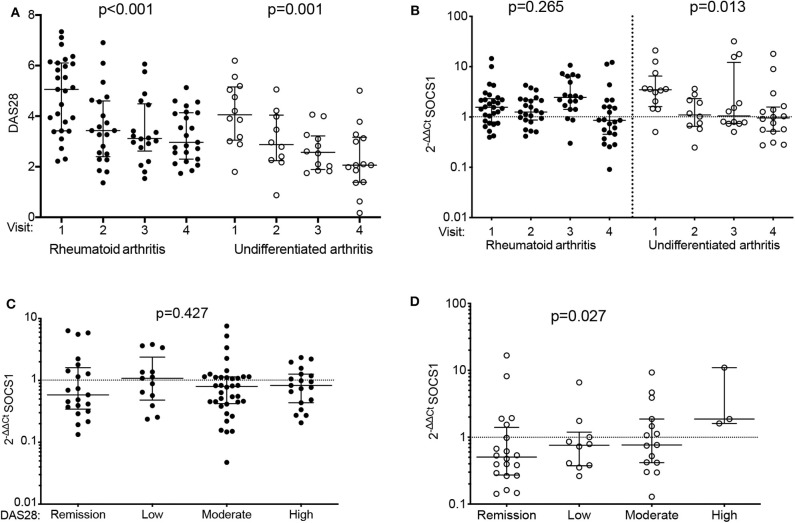
Differential *SOCS1* expression throughout follow-up of patients with early arthritis by final diagnosis: discovery population. **(A)** Evolution of disease activity by diagnosis (rheumatoid arthritis [RA]: solid dots; undifferentiated arthritis [UA]: empty dots). **(B)** Variation of *SOCS1* expression throughout follow-up in patients with RA (solid dots) or UA (empty dots). **(C,D)** *SOCS1* expression according to disease activity (DA) assessed by DAS28 in visits of patients with RA **(C)** or UA **(D)**. Data shown for mRNA *SOCS1* levels normalized to *ACTB* and to mean *SOCS1* expression levels in healthy donors (2^−ΔΔCt^). Error bars show medians and interquartile range. Statistical significance was determined with Cuzick's non-parametric trend test. RA patients, *n* = 70; UA patients, *n* = 34; healthy donors, *n* = 40.

Using blood samples collected at different visits (*n* = 143 total), we studied *SOCS1* expression in 104 patients from the PEARL register (discovery group; see Supplementary Table 2 for baseline characteristics). Despite a significant decrease in disease activity as a result of treatment adjusted over time (Figure 1A, Supplementary Table 3), we observed a significant reduction in *SOCS1* mRNA expression during follow-up of patients with UA (Figure 1B; *p* = 0.013), but no significant changes in *SOCS1* mRNA expression in patients with RA. When patients were classified by the DAS28 score (remission, low, moderate or high activity) and after pooling all visits, those with RA showed no difference in *SOCS1* expression (Figure 1C), whereas a significant direct correlation between *SOCS1* levels and disease activity was observed for patients with UA (Figure 1D; *p* = 0.027).

In light of these observations, we replicated the study in a second PEARL group engaged in the 2011–2014 period. This patient group used more stringent criteria, and included only those patients with definite RA classification according to the 1987 criteria (17), and for whom sample availability included baseline and ≥2 visits (validation group; *n* = 39, 111 samples; see Supplementary Table 2 for baseline characteristics). Analysis of these samples confirmed a decrease in disease activity (Supplementary Figure 1A) that did not correlate with a reduction in *SOCS1* expression either when patients were classified by follow-up visit number (Supplementary Figure 1B), by DAS28 score, (Supplementary Figure 1C), or when considered globally (Supplementary Figure 1D).

We detected some significant differences between the discovery and validation groups for sex, diagnosis, rheumatoid factor (RF) and antibodies to anti-citrullinated proteins (ACPA), likely due to the relatively low number of patients with definite RA in the validation group (Supplementary Table 2). As these variables could act as confounders in the analysis, we reanalyzed the pooled data using a longitudinal multivariate model nested by patients and visits. *SOCS1* expression was significantly lower in patients older than 65 years, as well as in those with increased hemoglobin levels (Table 2). Glucocorticoids treatment and patients with UA tended to associate with higher *SOCS1* expression (Table 2). The model adjusted by the aforementioned confounders showed significantly lower *SOCS1* mRNA levels in samples from visits with moderate or high disease activity compared with those of patients in remission (Table 2). The correlation of *SOCS1* values with those variables included in the multivariable analysis (age, glucocorticoids, disease activity and hemoglobin) is shown in Supplementary Figures 2A–D, and information on *SOCS1* levels in RA vs. UA is included in Supplementary Figure 2E.

**Table 2:** Variables that affect *SOCS1* expression: multivariate analysis performed in the pooled PEARL population.

**Variable**	**Value**	**β-coefficient**	**Standard error**	***p*-value**
Age at disease onset (years)	<45	Reference	–	–
	45–65	0.96	0.93	0.306
	>65	−10.28	3.07	0.001
Hemoglobin (gr/dl)		−6.44	1.75	<0.001
Glucocorticoids treatment	No	Reference	–	–
	Yes	2.23	1.42	0.116
Diagnosis	Rheumatoid arthritis	Reference	–	–
	Undifferentiated arthritis	7.27	7.55	0.336
Disease activity	Remission	Reference	–	–
	Low	−6.70	14.31	0.640
	Moderate	−14.98	3.92	<0.001
	High	−6.93	1.59	<0.001

**Figure 2:**
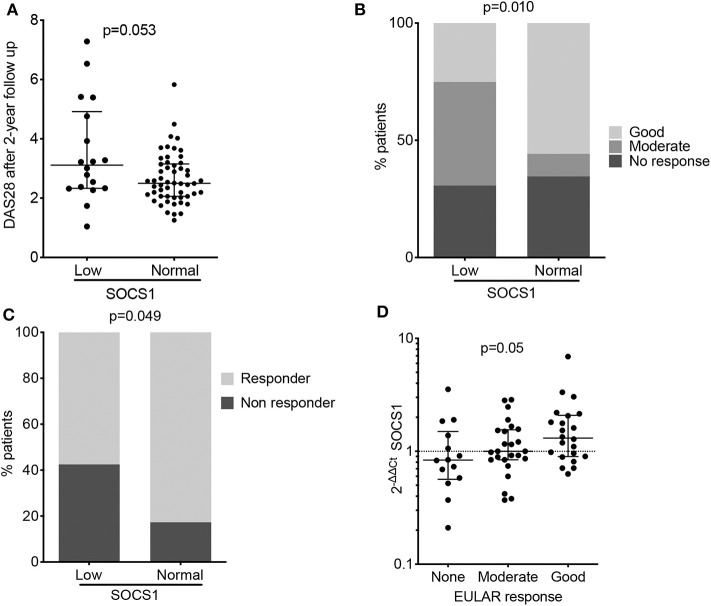
Baseline *SOCS1* expression as a severity biomarker in patients with RA (PEARL study). **(A)** Disease activity estimated by DAS28 score after 2 years of follow-up, relative to baseline *SOCS1* expression levels. Data shown as individual values with median and interquartile range. Statistical significance was established by the Mann-Whitney U test; low *SOCS1* levels (*n* = 25) were defined as those values below percentile 25 at the baseline of PEARL population; the remaining patients were considered to have a normal *SOCS1* level (*n* = 67). **(B)** Percentage of patients classified by EULAR response criteria after 12 months follow-up in PEARL subpopulations. Statistical significance was determined by the χ2 test. low SOCS1 levels (*n* = 25) were defined as those values below percentile 25 at the baseline of PEARL population; the remaining patients were considered to have a normal SOCS1 level (*n* = 67) as described for panel **(A)**. **(C)** Percentage of responder and non-responder patients after 6 months rituximab infusion. Low *SOCS1* levels (*n* = 14) were defined as those values below percentile 25 at the baseline of the Leeds established RA population; the remaining patients were considered to have a normal *SOCS1* levels (*n* = 48). Statistical significance was determined as in **(B)**. **(D)** Baseline *SOCS1* expression relative to EULAR response criteria after 6-months rituximab infusion (Leeds study). Data shown for mRNA *SOCS1* levels normalized to *ACTB* and to mean *SOCS1* expression levels in healthy donors (2^−ΔΔCt^); error bars show medians and interquartile range; (*n* = 14 no response, *n* = 25 moderate response and *n* = 23 good response). Statistical significance was determined using Cuzick's non-parametric trend test.

We hypothesized that patients with low *SOCS1* expression and high disease activity at baseline might coincide with those unable to achieve adequate control of disease at follow-up. We thus tested whether low *SOCS1* levels at baseline (values below the 25th percentile) were associated with higher disease activity after 2 years. In the combined PEARL patient subsets (*n* = 143), our analysis showed a trend for higher disease activity after 2 years in patients with low *SOCS1* expression at baseline (Figure 2A, *p* = 0.053). The lack of significance was not unexpected given the heterogeneity of the cohorts evaluated. PEARL is a longitudinal observational register and includes patients with different treatments and even with intensified treatments in cases when the disease was not under control.

### *SOCS1* Expression and Response to Therapy

Disease activity, a parameter strongly influenced by patient treatment, was heterogeneous throughout the PEARL follow-up (Supplementary Table 3). We nevertheless evaluated the potential of baseline *SOCS1* expression to predict EULAR response at 6-, 12-, and 24-months of follow-up. Responder patients with lower *SOCS1* mRNA levels at baseline showed poorer EULAR response rates in the three visits, although this was statistically significant only at the 12-month visit (Figure 2B; *p* = 0.010).

Data from the independent early, drug-naïve, Leeds RA cohort (*n* = 74) treated according to the treat-to-target protocol (i.e., the achievement of remission (24) with synthetic DMARDs (methotrexate dose escalation) confirmed that low *SOCS1* baseline expression was associated with inability to achieve remission at 6 months (Table 3, *p* = 0.036). Receiver operating characteristic curve analysis (Supplementary Figure 3) demonstrated an area under the curve of 0.644 (95% confidence interval 0.514–0.775) for predicting remission. A regression analysis (Supplementary Table 4) showed the best predictive model (achieving 73% accuracy) used 4 variables (Age, TJC, CRP and *SOCS1*) although replacing TJC/CRP by DAS28 in a second model improved the regression but had no effect on accuracy. An AUROC analysis of the variables retained in the regression (Supplementary Table 5) showed that SOCS1 was the second best predictor of remission after TJC in this small early RA cohort (sensitivity/specificity 56%/67%, odd ratio 1.73, and positive/negative predictive value 78%/64%).

**Table 3:** Baseline characteristics of the early RA LEEDS population studied.

**mtx**	**Remission (*n =* 29)**	**No remission (*n =* 45)**	***p*-value**
Age^*^ (years)	59 (47–66)	50 (41–62)	0.408
Sex (M/F)	7/22	11/34	0.601
Smoking	15/14	16/28	0.231
Symptoms (m)^*^	6.2 [4.8–12.9]	6 (3.7–13.6)	0.829
RF (+/–)	15/14	22/22	0.538
ACPA (+/–)	20/9	23/22	0.129
TJC^*^	2 (1–10)	10 (4–15)	**0.001**
SJC^*^	3 (0–9)	4 (1–8)	0.587
CRP^*^	8 (<5–18.5)	12 (<5–27)	0.077
DAS28^*^	4.10 (2.24–4.80)	4.50 (3.40–5.6)	**0.020**
SOCS1^*^^†^	0.41 (0.21–0.58)	0.26 (0.08–0.47)	**0.036**
**RITUXIMAB**	**No response (*****n=*** **16)**	**Mod/Good response (*****n=*** **49)**	***p*****-value**
Age^*^ (years)	48 (46–64)	57 (48–64)	0.598
Sex (M/F)	2/14	9/40	0.443
symptoms (m)^*^	70 (25–94)	102 (36–204)	0.244
RF (+/–)	16/0	42/7	0.123
ACPA (+/–)	15/1	46/3	0.687
TJC^*^	15 (9–24)	16 (10–23)	0.703
SJC^*^	7.5 (3–17)	8 (5–11)	0.328
CRP^*^	46 (>5–123)	12.5 (>5–23)	0.192
DAS28^*^	5.76 (4.6–7.2)	5.67 (4.7–6.2)	0.732
SOCS1^*^^†^	0.640 (0.273–0.775)	0.880 (0.680–1.405)	**0.035**

M, male; F, female; RF, rheumatoid factor; ACPA, anti-citrullinated proteins antibodies; TJC, tender joint count; SJC, swollen joint count; CRP, C-reactive protein; DAS28, disease activity score calculated with the 28 joint count; SOCS1, suppressor of cytokine signaling;

† (normalized quantity, using GADPDH)

* *median and (interquartile range). Values in bold are statistical significance*.

The association between *SOCS1* expression and response to treatment was further investigated with the 64 patients from Leeds with established RA who were treated with rituximab (Table 3). This analysis revealed only one significant association between non-response to treatment and low baseline *SOCS1* levels in this small cohort (Figure 2C, *p* = 0.049). Baseline *SOCS1* levels also differed significantly depending on EULAR response classification 6-months post-rituximab treatment (Figure 2D; *p* = 0.050).

### The rs4780355 Polymorphism in the *SOCS1* Sequence Associates With Reduced *SOCS1* Expression

Given the potential clinical relevance of determining *SOCS1* mRNA levels in early RA, we tested whether genetic variability in the *SOCS1* gene and adjacent areas influences *SOCS1* expression and clinical parameters. Immunochip data (25) extracted for SNPs near the *SOCS1* gene and also located at the two intergenic regions gave a total of 47 SNPs. We generated a linkage disequilibrium plot using Haploview software and defined three haplotype blocks of 2, 4 and 12 kb (Supplementary Figure 4).

After linkage disequilibrium analysis, 10 SNPs representative of the genetic variability in the *SOCS1* gene and adjacent areas (rs11074956, rs181582, rs149597, rs2021760, rs4780355, rs193779, rs243327, rs1559392, rs3844576, and rs243323) were selected for subsequent genotyping in PEARL patients using predesigned TaqMan probes. The 10 SNPs were studied for association with baseline low *SOCS1* mRNA, RA diagnosis at the end of follow-up, and response to treatment at 12 months of follow-up (Supplementary Table 6). The minor allele of four of these SNPs tended to associate with low *SOCS1* expression; and another two SNPs with high levels at baseline (*p* < 0.150; Supplementary Table 6). We observed that from the four SNPs whose minor allele was associated with low *SOCS1* expression, two were also linked to a higher odds ratio of RA diagnosis (*p* < 0.150), and one of these two (rs4780355) was linked to poor response (*p* < 0.150) (Supplementary Table 6), suggesting that this SNP might predict a poorer clinical course in patients with early arthritis. Regarding the relaxed *p*-values considered in these analyses, it should be mentioned that several SNPs in addition to rs4780355 might affect *SOCS1* expression, limiting the feasibility of recruiting a sufficient number of patients per subgroup to allow the analysis of *SOCS1* expression by haplotypes.

Both rs11074956 and rs4780355 have been associated with some autoimmune and inflammatory diseases (26, 27), but we focused on rs4780355 (T/C *SOCS1*) because it was the most consistent with the considered criteria (Supplementary Table 6), and it is located near the *SOCS1* 3′ untranslated region (UTR) (Supplementary Figure 5), allowing the study of its influence on *SOCS1* expression in *in vitro* transcription assays.

**Figure 3:**
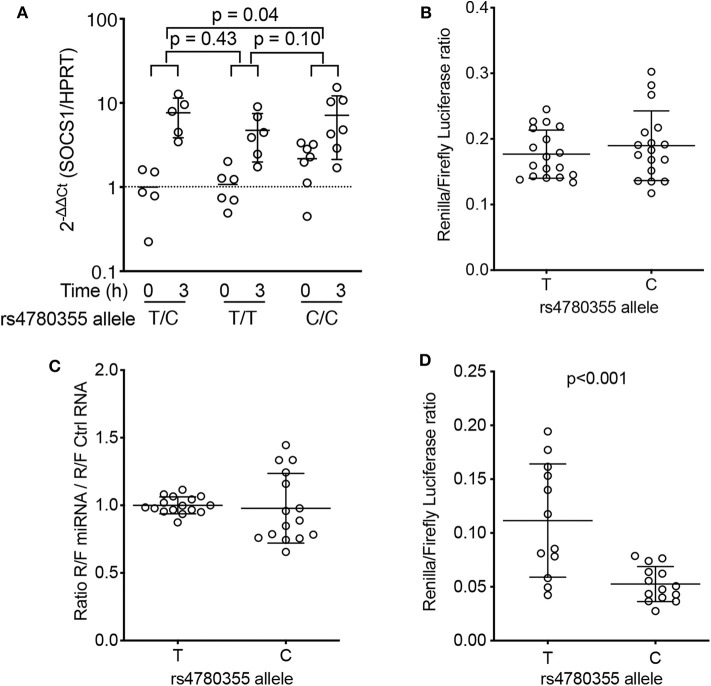
Effects of rs4780355-linked genomic DNA features on *SOCS1* expression. **(A)** Peripheral blood mononuclear cells from 18 patients with RA (five T/C heterozygous, seven C/C homozygous and six T/T homozygous for rs4780355) were PHA-activated for 3 h. *SOCS1* levels were determined by qRT-PCR (see Methods) before and after 3 h treatment. Values (2^−ΔΔCt^) are normalized to *ACTB* and the mean value from heterozygous samples at t = 0; *p*-values from unpaired *t*-tests are indicated. **(B)** Luciferase assays. Two independent constructs for each genotype, which included the *SOCS1* polyadenylation site and rs4780355 SNP, were used for Jurkat cell transfection; the *Renilla*/Firefly luciferase ratio was measured 24 h later. **(C)** miR-498 or control RNA (MISSION® siRNA Universal Negative Control #2, Sigma) were co-transfected with plasmid constructs. *Renilla*/Firefly ratios obtained with miR-498 were normalized to control miRNA values. Results from two independent clones and triplicate measurements. **(D)** Two independent clones, including the polyadenylation site, rs4780355 SNP (C or T) and its associated microsatellite [[TTTTC]_3_ or [TTTTC]_5_, respectively], were tested for each haplotype in two experiments with triplicate samples. Statistical significance was determined by the Mann-Whitney U test.

To begin to examine the implications of rs4780355 (T/C *SOCS1*) for *SOCS1*, we first isolated PBMCs from homozygous C/C and T/T, and heterozygous C/T patients, and measured *SOCS1* mRNA levels before and after *in vitro* activation of cells with PHA. PHA stimulation mimics T-cell receptor activation (28), and therefore does not restrict the analysis to a single cytokine, thus amplifying the possibilities of an effect triggered by SOCS1. Although *SOCS1* is expressed in control conditions (29), its expression is upregulated following cell activation (13). Results showed that *SOCS1* mRNA induction in PHA-activated PBMCs was lower for homozygous (C/C) patients than for heterozygous (C/T) patients (*p* = 0.048), and there was also a trend for lower expression when compared with homozygous (T/T) patients (*p* = 0.1) (Figure 3A). Hematological analysis of these patients showed no differences in the numbers of lymphocytes or monocytes (Supplementary Figures 6A,B).

We next used an *in vitro* system to examine the role of this polymorphism in *SOCS1* expression. The *SOCS1* 3′UTR (containing the T or C allele region) was cloned into the psiCHECK-2 vector (see Methods). Jurkat cell transfection assays showed no significant differences in the ratio of *Renilla*/Firefly luciferase between constructs with T or C alleles (four independent clones for each allele; Figure 3B). Analysis of the miRDB database (www.mirdb.org) pointed to the DNA region containing the rs4780355 polymorphism as a potential target for the microRNA miR-498 (Supplementary Figure 7). This microRNA is downregulated in CD4^+^ T-cells in patients with RA (30), which suggests a role in RA pathogenesis. We therefore transfected Jurkat cells with the luciferase constructs for each allele in the presence of miR-498 or an miR-control. Data showed no significant difference in luciferase activity between the two conditions (Figure 3C), indicating the lack of a direct effect of the polymorphism or a potential role for miR-498 in *SOCS1* expression.

Detailed analysis of the DNA sequence surrounding the rs4780355 polymorphism identified a microsatellite followed by a variable T-string, GCT[TTTTC]_(3−5)_T_(11−19)_GC, 0.9 kbp downstream of the polymorphism. The observed sequence variations were deposited in NCBI's ClinVar database SCV000999906 (Supplementary Figure 8). In Spanish and British populations, allele C is linked (*R*^2^ = 0.9456) to the short microsatellite (three repeats), whereas allele T is associated with the long form (five repeats) (Supplementary Figure 9). We thus generated a construct containing the *SOCS1* 3′UTR (including the T or C allele region and the associated microsatellites) cloned into the psiCHECK-2 vector. Jurkat cell transfection showed a significant reduction in the *Renilla*/Firefly luciferase ratio when the construct contained the C allele, that is, the short form of the microsatellite (Figure 3D). These data suggest that the effect of rs4780355 on *SOCS1* expression is related to presence of the microsatellite (TTTTC)_3−5_ and the variable T-string in the 3′UTR region.

## Discussion

Genome-wide association studies have found multiple SNPs on genes that encode cytokines, their receptors and/or signaling molecules, associating with inflammatory and autoimmune disease risk (31, 32). This is also the case for RA, an autoimmune disease for which cytokine-based therapies are included in the armamentarium of drugs for the treatment of patients with an inadequate response to conventional DMARDs (33–36). Baricitinib and tofacitinib, two small-molecule JAK inhibitors that block JAK/STAT signaling, have recently been approved for RA treatment (7). Physiologically, cytokine activation also triggers downregulatory mechanisms that protect cells from hyperactivation. Cytokines promote the expression of SOCS proteins that not only block JAK/STAT signaling, but also trigger protein degradation by the proteasome (37). Here we show that patients with early arthritis who are unable to upregulate *SOCS1* expression are more likely to be classified as having RA and have a higher risk of not achieving remission and/or failing to respond to treatment. Our data concur with previous observations showing that *Socs1*^−/−^ mice are hypersusceptible to IL-1-mediated acute inflammatory arthritis and have increased joint damage, with no change in the time course of arthritis (38). In a similar line, *Stat1*^−/−^ mice show exacerbated zymosan-induced arthritis, possibly due to reduced SOCS1 expression (39). Ectopic expression of SOCS1 abolishes IFN-β-mediated STAT1α stimulation and prevents IFN-β-induced expression of CD40 (40), a protein involved in RA pathogenesis (7). In addition to its effect on the JAK-STAT pathway, SOCS1 interacts with the adaptor protein GRB2, the phosphoprotein VAV, calcineurin, and IL-1 receptor-associated kinase, and thereby suppresses signaling through KIT, T-cell receptor, and Toll-like receptors 2 and 4 (41, 42). SOCS1 inhibits MYD88 adaptor-like protein degradation and thus regulates NF-kB activation and, accordingly, *Socs1*^−/−^ mice show increased susceptibility to chronic LPS-induced inflammation (43, 44). This pathway is involved in the expression of several inflammatory mediators, including TNF-α, IL-1β, IL-6, and type I interferon, all useful targets for biological therapy for RA (45, 46).

In humans, SOCS1 expression is associated with CD4^+^ T-cell resistance to the immunosuppressive effect of IL-10, which is detected before the cells migrate to synovial tissue (47). Expression of miR-155, a microRNA that targets *SOCS1*, has been detected in PBMCs from patients with RA, and is involved in TNFα and IL-1β upregulation (48). Correlation between DNA methylation of *SOCS1* and the degree of inflammation, assessed by TNFα and IL-6 levels, was also shown in patients with HLA-B27^+^ spondylitis (49).

Non-activated immune cells express low levels of *SOCS*, which is rapidly and transiently upregulated after cytokine activation (13). We found that patients with UA in remission had low levels of *SOCS1* that increased in parallel to disease activity. By contrast, *SOCS1* mRNA levels did not correlate with disease activity in patients with RA, but those with the lowest baseline levels showed the poorest clinical evolution. Genetic variants that affect *SOCS1* expression thus might alter the course of RA. In our analysis, several *SOCS1* gene variants affected its mRNA levels. Three SNPs (rs11074956, rs4780355, and rs243323) were associated with low *SOCS1* expression and a tendency for poorer clinical outcomes. In an *in vitro* luciferase-based assay, we detected lower *SOCS1* expression in Jurkat cells expressing the minor allele of rs4780355. This SNP is located in Chr16:11254001 (GRCh38.p7) within the 3′UTR of the *SOCS1* gene. *In silico* analysis indicated that the *SOCS1* sequence involving the rs4780355 polymorphism is a potential target for miR-498. Interestingly, miR-498 directly targets the 3′UTR of *STAT3* and is downregulated in CD4^+^ T-cells of patients with RA (30). We hypothesized that it might also target the *SOCS1* 3′UTR region, triggering *SOCS1* downregulation and thus cytokine hyperactivation. However, based on our *in vitro* luciferase-based assays using cells transfected with miR-498, we discarded this hypothesis.

We found that rs4780355 is in linkage disequilibrium with expansions of simple sequence repeats, microsatellites, in a non-coding region near the *SOCS1* 3′UTR. In the population origin of our cohorts, the C allele is linked to the shortest form of the microsatellite, whereas the T allele is associated with five repeats. The *in vitro* luciferase-based assays linked the C allele to reduced *SOCS1* expression when cells bore the minor allele of rs4780355. RNA misprocessing has been reported for a number microsatellite-expansion diseases (50), and in other cases microsatellites cause gene silencing, possibly due to impairment of transcriptional elongation (51). RNA polymerase II transcription frequently terminates 500 or more bp downstream of the poly(A) signal (52). Intron or UTR retention during RNA processing could affect nuclear retention, nucleocytoplasmic transport, and cytoplasmic turnover and is a conserved regulatory mechanism that affects a wide range of cellular events (53, 54). Our luciferase-based assays suggest that the presence of the shorter microsatellite influences mRNA processing and thus promotes reduced protein expression. Although more experiments are needed to clarify the mechanism, we speculate that it involves RNA stability and/or transcriptional termination efficiency.

The current paradigm of RA management holds that disease is better controlled when very early DMARD treatment is established (55). New lines of research indicate that early treatment of patients is associated with better disease outcomes (56); however, the clinical spectrum of RA is very heterogeneous and the discovery of new severity biomarkers will be needed to establish tailored treatments. Our data suggest that measuring *SOCS1* mRNA levels could be a technically reliable and robust procedure with which to predict those patients who will have a poorer clinical evolution. This is supported by data obtained in the discovery population, validated in a separate group of patients selected using more stringent RA criteria, and replicated in two independent populations for early and established RA.

Our observations, nonetheless, indicate some limitations to implementing the use of this biomarker, including determining the appropriate baseline low levels of *SOCS1*. Prior adjustment by age and glucocorticoid use is also needed to allow efficient patient classification. In addition, *SOCS1* expression did not discriminate between patients in remission regarding pre-arthritis stages or treatment effects. Genotyping of rs11074956, rs4780355, and rs243323, whose minor alleles were associated with lower *SOCS1* baseline levels, might overcome these limitations.

Some authors propose that personalized medicine for RA is currently not possible (57), and data from daily clinical practice suggest that earlier treatment for inflammation improves clinical outcome (5). Our findings point to new candidates for the development of composite biomarker indices with greater predictive ability for RA severity that could be useful to guide patient care from establishment of the diagnosis.

## Data Availability Statement

The datasets generated for this study can be found in the ClinVar, SCV000999906.

## Ethics Statement

The studies involving human participants were reviewed and approved by The PEARL study was approved by the local Research Ethics Committee (PI-518). All patients gave informed consent. The Leeds cohorts approval was obtained from local Research Ethics Committees for both groups (REC: 07/S0703/68, 10/H1307/138). Informed consent was obtained from all individuals. The patients/participants provided their written informed consent to participate in this study.

## Author Contributions

AL, RV, IS, NA, PL, CM, and RG performed the experiments. IS, AM, and JM analyzed the genetic variability of *SOCS1* gene from Immunochip data. PE, EV, RM, and FP recruited the patients of the Leeds cohorts and obtained the samples. AT-M, AO, and IG-A recruited the patients of the PEARL study and obtained the samples. RV, FP, and IG-A performed the statistical analysis. RV, IG-A, and MM wrote the manuscript. IG-A and MM conceived the study. All authors contributed to the article and approved the submitted version.

## Conflict of Interest

The authors declare that the research was conducted in the absence of any commercial or financial relationships that could be construed as a potential conflict of interest.
