# Supplementary material for: Identification of a Human SOCS1 Polymorphism That Predicts Rheumatoid Arthritis Severity
Source: Front Immunol. 2020 Jun 26;11:1336. doi: 10.3389/fimmu.2020.01336 (PMC7332777; doi:10.3389/fimmu.2020.01336)
Supplement: Supplementary file 1 [file Data_Sheet_1.docx]

**SUPPLEMENTARY INFORMATION**

**Supplementary Table 1. Characteristics of PEARL subpopulations included (Studied) or excluded (Not Studied) in this study**

|  | Not Studied  (n=313) | Studied  (n=143) | p-value |
| --- | --- | --- | --- |
| Female; n (%) | 243 (78) | 118 (82) | 0.234 |
| Age; p50 [p25-p75] | 55 [43 – 66] | 54 [45 – 69] | 0.430 |
| RA / UA; n (%) | 219 (70) / 94 (30) | 106 (74) / 37 (26) | 0.363 |
| Disease duration (months); p50 [p25-p75] | 5.6 [3.1 – 8.8] | 5.3 [3.0 – 8.4] | 0.437 |
| RF positive; n (%) | 162 (52) | 81 (57) | 0.332 |
| ACPA positive; n (%) | 147 (48) | 73 (51) | 0.449 |
| DAS28; p50 [p25-p75] | 4.4 [3.3 – 5.6] | 4.8 [3.4 – 5.7] | 0.208 |
| HAQ; p50 [p25-p75] | 0.875 [0.5 – 1.625] | 1 [0.625 – 1.625] | 0.089 |

n: number; p50: median or percentile 50; p25-p75: interquartile range; RA: rheumatoid arthritis; UA: undifferentiated arthritis; RF: rheumatoid factor; ACPA: anti-citrullinated protein antibodies; DAS28: disease activity score estimated with the 28 joint count; HAQ: health assessment questionnaire

**Supplementary Table 2. Characteristics of discovery and validation PEARL subpopulations studied**

|  | Discovery (n=104) | Validation (n=39) | p-value |
| --- | --- | --- | --- |
| Female; n (%) | 82 (79) | 36 (92) | 0.059 |
| Age; p50 [p25-p75] | 56 [46 – 70] | 51 [39 – 67] | 0.138 |
| RA / UA; n (%) | 70 (67) / 34 (33) | 39 (100) | <0.001 |
| Disease duration (months); p50 [p25-p75] | 5.1 [3.0 – 7.7] | 6.2 [2.7 – 10.1] | 0.349 |
| RF positive; n (%) | 51 (49) | 30 (77) | 0.003 |
| ACPA positive; n (%) | 48 (47) | 25 (64) | 0.063 |
| DAS28; p50 [p25-p75] | 4.8 [3.4 – 5.8] | 4.9 [3.5 – 5.6] | 0.946 |
| HAQ; p50 [p25-p75] | 1 [0.75 – 1.625] | 1 [0.375 – 1.5] | 0.293 |

n: number; p50: median or percentile 50; p25-p75: interquartile range; RA: rheumatoid arthritis; UA: undifferentiated arthritis; RF: rheumatoid factor; ACPA: anti-citrullinated protein antibodies; DAS28: disease activity score estimated with the 28 joint count; HAQ: health assessment questionnaire

**Supplementary Table 3. Treatments prescribed throughout follow-up for discovery and validation PEARL subpopulations**

|  | Discovery population | | |
| --- | --- | --- | --- |
|  | 6 months | 12 months | 24 months |
| Methotrexate (%) | 60.6 | 61.3 | 48.7 |
| Antimalarial (%) | 12.1 | 6.4 | 12.8 |
| Leflunomide (%) | 24.2 | 19.4 | 25.6 |
| Sulphasalacine (%) | 3.0 | 0.0 | 7.7 |
| Gold salts (%) | 0.0 | 0.0 | 2.6 |
| Biological therapy (%) | 0.0 | 16.1 | 0.0 |
| Prednisone (%) | 66.7 | 58.1 | 35.9 |
|  | Validation population | | |
|  | 6 months | 12 months | 24 months |
| Methotrexate (%) | 81.5 | 65.2 | 64.7 |
| Antimalarial (%) | 7.4 | 8.7 | 5.9 |
| Leflunomide (%) | 14.8 | 26.1 | 35.3 |
| Sulphasalacine (%) | 3.7 | 4.4 | 5.9 |
| Gold salts (%) | 0.0 | 0.0 | 0.0 |
| Biological therapy (%) | 3.7 | 13.0 | 11.8 |
| Prednisone (%) | 55.6 | 39.1 | 47.1 |

**Supplementary Table 4. Regression models (both models had a 73% accuracy)**

|  | Unadjusted Odd ratio^$^ | 1^st^ Adjusted model^$^ | 2^nd^ Adjusted model^$^ |
| --- | --- | --- | --- |
| Age* (years) | 1.030 (0.99-1.07) 0.070 | 1.038 (0.99-1.08) 0.110 | 1.039 (0.99-1.08) 0.09 |
| Sex (M/F) | 1.070 (0.34-3.02) 0.976 |  |  |
| Symptoms (m)* | 0.977 (0.91-1.04) 0.500 |  |  |
| Smoking^&^ | 0.533 (0.20-1.38) 0.199 |  |  |
| RF (+/-) | 1.070 (0.42-2.72) 0.885 |  |  |
| ACPA (+/-) | 2.12 (0.789-5.66) 0.132 | 2 |  |
| TJC* | 0.906 (0.83-0.98) **0.015** | 0.893 (0.81-0.98) **0.014** |  |
| SJC* | 1.011 (0.93-1.11) 0.715 |  |  |
| CRP* | 0.977 (0.95-1.00) 0.098 | 0.975 (0.94-1.01) 0.125 |  |
| DAS28* | 0.701 (0.49-0.99) 0.049 |  | 0.645 (0.44-0.95) **0.028** |
| *SOCS1**† | 6.102 (1.38-27.13) **0.017** | 9.78 (1.50-60.60) **0.014** | 10.621 (1.75-65.5) **0.010** |

M: male; F: female; RF: rheumatoid factor; ACPA: anti-citrullinated proteins antibodies; TJC: tender joint count; SJC: swollen joint count; CRP: C-reactive protein; DAS28: disease activity score calculated with the 28 joint count; *SOCS1*: suppressor of cytokine signaling; ^&^ smoking reported as never/ever (missing data in 1 patient).

$ odd ratio (95% CI) p-value

*Median (interquartile range);

† (normalized quantity)

**Supplementary Table 5. Predictive values :**

|  | AUROC^£^ | Cut-off | Predictive values^€^ |
| --- | --- | --- | --- |
| Age* (years) | 0.623 (0.49-0.76) 0.072 | Over 60 | 50%/67% - 1.50 - 71%/61% |
| TJC* | 0.326 (0.20-0.45) **0.013** | Less/equal 3 | 58%/72% - 2.1 - 75%/67% |
| CRP* | 0.401 (0.27-0.53) 0.156 | Detectable (>5 mg/L) | 46%/67% - 1.39 - 67%/59% |
| DAS28* | 0.364 (0.23-0.50) 0.060 | Over 3.2 | 53%/68% - 1.67 – 79%/64% |
| *SOCS1**† | 0.664 (0.53-0.79) **0.019** | Over 0.50 | 56%/67% - 1.73 - 78%/64% |

TJC: tender joint count; CRP: C-reactive protein; DAS28: disease activity score calculated with the 28 joint count; *SOCS1*: suppressor of cytokine signaling;

AUROC (95% CI) p-value.

^€^ sensitivity/specificity –OR - PPV/NPV.

† (normalized quantity)

**Supplementary Table 6. Effect of the minor allele of 10 selected SNPs in *SOCS1* and adjacent areas on variables for PEARL patients studied**

| SNP | Alleles | MAF in PEARL  n = 456 | Low *SOCS1* at baseline*  (OR ± SE)  n=92 | p | RA diagnosis at end of  F-U (OR ± SE)  n=456 | p | Response at month 12**  (OR ± SE)  n=379 | p |
| --- | --- | --- | --- | --- | --- | --- | --- | --- |
| rs11074956 | G/**T** | 33.6% | 2.6 ± 1.2 | 0.035 | 1.3 ± 0.2 | 0.140 | 0.9 ±0.1 | 0.366 |
| rs181582 | C/**T** | 21.6% | 0.8 ± 0.4 | 0.603 | 0.8 ± 0.2 | 0.344 | 1.1 ± 0.2 | 0.710 |
| rs149597 | C/**G** | 47.5% | 0.6 ± 0.2 | 0.149 | 0.8 ± 0.1 | 0.075 | 1.1 ± 0.2 | 0.459 |
| rs2021760 | A/**G** | 12.8% | 1.3 ± 0.7 | 0.683 | 1.7 ± 0.4 | 0.024 | 0.8 ± 0.2 | 0.276 |
| rs4780355 | T/**C** | 35.6% | 2.0 ± 0.8 | 0.068 | 1.2 ± 0.2 | 0.147 | 0.8 ± 0.1 | 0.127 |
| rs193779 | G/**A** | 26.8% | 1.1 ± 0.5 | 0.890 | 1.0 ± 0.2 | 0.999 | 0.9 ± 0.2 | 0.501 |
| rs243327 | T/**C** | 49.2% | 1.9 ± 0.8 | 0.107 | 1.1 ± 0.2 | 0.472 | 1.0 ± 0.1 | 0.734 |
| rs1559392 | C/**T** | 23.7% | 0.3 ± 0.2 | 0.125 | 0.8 ± 0.2 | 0.327 | 1.2 ± 0.2 | 0.343 |
| rs3844576 | G/**T** | 14.0% | 1.7 ± 0.8 | 0.256 | 1.2 ± 0.3 | 0.299 | 0.5 ± 0.1 | 0.001 |
| rs243323 | A/**G** | 31.8% | 1.9 ± 0.8 | 0.092 | 1.1 ± 0.2 | 0.378 | 0.8 ± 0.1 | 0.084 |

*SOCS1*: Suppressor of cytokine signaling 1; PEARL study: Princesa Early Arthritis Register Longitudinal study; SNP: single nucleotide polymorphism; MAF: minor allele frequency; OR: odds ratio; SE: standard error; F-U: follow-up. Minor alleles in bold.

Shadowed cells mark a significance level p<0.15 for the association of SNPs and clinical variables.

* Adjusted by age, diagnosis, disease activity level, glucocorticoid use and hemoglobin

** Adjusted by age and gender

**Supplementary Figure 1. *SOCS1* expression throughout follow-up of early arthritis in patients from the PEARL validation population.** A) Improvement in disease activity throughout follow-up. B) *SOCS1* mRNA levels during follow-up. C) *SOCS1* gene expression according to disease activity level, assessed by DAS28 in the follow-up visits. A-C, data for *SOCS1* mRNA levels shown as interquartile range (p75 upper box edge, p25 lower edge, p50 midline), p90 (line above box) and p10 (line below). Dots represent outliers. Statistical significance was determined with Cuzick’s non-parametric trend test. D) Lack of correlation between *SOCS1* gene expression and DAS28 values. Dots indicate individual visits. Black line, linear regression prediction determined using the Stata *twoway* command with the *lfit* option*.* CC: correlation coefficient. p-value was obtained using Pearson’s correlation test.

**Supplementary Figure 2. Correlation line graphs of *SOCS1* expression values with those variables included in the multivariate analysis.** Correlation for age (A), glucocorticoid treatment (B), disease activity (C) and blood hemoglobin levels (D) against 2^-∆Ct^ *SOCS1* levels normalized to *ACTB.* Grey shadows represent 95% confidence index estimated for each variable with the *twoway fpfitci* command of Stata 14. A boxplot with interquartile range information (E) compares *SOCS1* levels in RA *vs* undifferentiated arthritis (UA); no significant difference is observed.


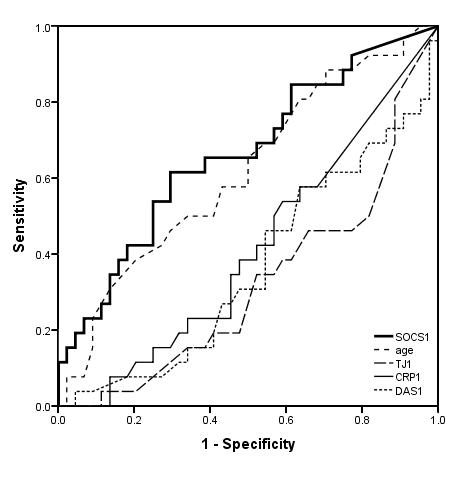


**Supplementary Figure 3.** ROC curves for the ability of different baseline variables (SOCS1 levels, age, tender joint count [TJC], C-reactive Protein [CRP] or disease activity score with 28 joint counts [DAS28]) to discriminate between achivement of remission or not in patients with early arthritis treated with methotrexate.

**Supplementary Figure 4. Linkage disequilibrium (LD) plot of the 47 SNPs studied in the *SOCS1* gene and adjacent regions.** LD plot of common variants (minor allele frequency threshold 0.01) in the *SOCS1* region based on genotyping data extracted from iCHIP. Plot shows D' (normalized linkage disequilibrium measure or D) values. LOD was defined as log_10_(L1/L0), where L1 = likelihood of the data under linkage disequilibrium, and L0 = likelihood of the data under linkage equilibrium. D' was calculated as follows: D' = (D) divided by the theoretical maximum for the observed allele frequencies. Red blocks, D' ≤1.0, with logarithm of odds (LOD) score ≥2.0; white blocks, D' <1.0 with LOD <2.0; blue blocks, D' =1.0 with LOD <2.0. Numbers in blocks denote D' values. The genomic organization is described above the LD plot; the *SOCS1* gene was located between rs33932899 and rs33977706 (Chr16: 11348274..11350039). Black arrows show the positions of markers genotyped in this study.

**
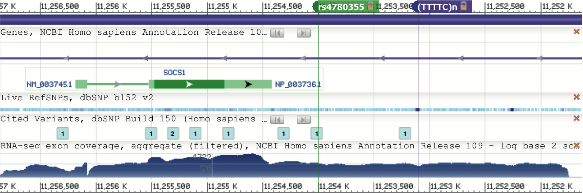
**

**Supplementary Figure 5.** **Genomic organization and chromosome location of human *SOCS1*, SNP rs4780355 and its associated microsatellite.** Public RNA-seq data are included.

**Supplementary Figure 6. Hematological analysis to determine the numbers of lymphocytes (A) and monocytes (B) in the blood samples from homozygous C/C and T/T, and heterozygous C/T RA patients**. Data show no statistical differences associated with the rs4780355 genotype.

**Supplementary Figure 7. MicroRNA-498 (miR) sequence and its putative binding site in the 3′ untranslated region of the *SOCS1* gene near rs4780355.** Alignment between miRNA-498 seed sequence (bold) and the two alleles of rs4780355 in *SOCS1* mRNA (bold italic).

**Supplementary Figure 8**. **DNA sequence in the region of TTTTC repeats found in 14 haploid isolates from healthy blood donors.** The corresponding rs4780355 allele is indicated in parentheses after the clone number. Consensus sequence as found in *Homo sapiens* Annotation Release 108 is included.

**Supplementary Figure 9. Linkage analysis of *SOCS1* 3’UTR.** CEU, GBR and IBS populations gathered from the 1000 Genomes database were analyzed online with LDlink. tools (<https://ldlink.nci.nih.gov>). SNP rs1111186 was selected as representative of the SNP cluster to which TTTTC repeats map.
